# Electro‐nape‐acupuncture regulates the differentiation of microglia through PD‐1/PD‐L1 reducing secondary brain injury in acute phase intracerebral hemorrhage rats

**DOI:** 10.1002/brb3.3229

**Published:** 2023-08-23

**Authors:** Yijian Liu, Shumei Zheng, Xiaohui Zhang, Wenhui Guo, Ruosang Du, Hongwen Yuan, Lu Zhang, Hai Cui

**Affiliations:** ^1^ School of Traditional Chinese Medicine, Capital Medical University Beijing China

**Keywords:** electro‐nape‐acupuncture, intracerebral hemorrhage, microglia differentiation, programmed cell death protein‐1

## Abstract

**Introduction:**

This study aimed to investigate the effect of electro‐nape‐acupuncture (ENA) on the differentiation of microglia and the secondary brain injury in rats with acute‐phase intracerebral hemorrhage (ICH) through the programmed cell death protein‐1/ligand‐1 (PD‐1/PD‐L1) pathway.

**Methods:**

A total of 27 male Sprague‐Dawley rats were randomly divided into three groups: sham group, ICH group, and ENA group. The autologous blood infusion intracerebral hemorrhage model was used to study the effects of ENA by administering electroacupuncture at GB20 (Fengchi) and Jiaji (EX‐B2) acupoints on 24 h after the modeling, once per day for 3 days. The neurological function damage, hematoma lesion, and inflammatory cell infiltration were measured by the beam walking test and hematoxylin‐eosin staining. The expression of PD‐1, PD‐L1, CD86, CD206, and related cytokines around the hematoma was measured by western blot, quantitative reverse transcription polymerase chain reaction, and immunofluorescence.

**Results:**

The ICH group had significant neurological deficits (*p* < .001), hematoma lesions, and inflammatory cell infiltration. The levels of CD86 protein, inflammatory factors tumor necrosis factors (TNF)‐α, interleukin (IL)‐1β, and IL‐6 were increased (*p* < .001), while CD206 protein was reduced (*p* < .01), and the number of CD86^+^/CD11b^+^ cells was also increased (*p* < .001) compared to the sham group. However, after ENA intervention, there was a significant reduction in neurological function damage (*p* < .05), infiltration of inflammatory cells, and the expression levels of CD86^+^/CD11b^+^ cells (*p* < .05), resulting in the increased expression of PD‐1 protein and differentiation of M2 phenotype significantly (*p* < .001).

**Conclusion:**

The study concludes that ENA could reduce neurological function damage, inhibit the expression of pro‐inflammatory cytokines, and improve the infiltration of inflammatory cells to improve secondary brain injury in acute‐phase intracerebral hemorrhage rats. These effects could be related to the increased expression of PD‐1 around the lesion, promoting the differentiation of microglia from M1 to M2 phenotype.

## Abstract 2

Electro‐nape‐acupuncture can reduce neurological function damage, inhibited the expression of pro‐inflammatory cytokines, and reduced the infiltration of inflammatory cells, thus improving the secondary brain injury.

## 1 INTRODUCTION

Intracerebral hemorrhage (ICH) is a devastating neurological disease that accounts for 8%–15% of strokes. It first changes to the rapid accumulation of blood in the brain, which increases intracranial pressure and compresses the brain tissue to make it ischemia and hypoxia, constituting primary brain injury (Felberg et al., 2002). In contrast, secondary injury caused by the toxic substances released from hematoma decomposition, oxidative stress, and activation of inflammatory responses (e.g., iron overload, activation of microglia/astrocytes, the release of inflammatory factors, etc.) plays a crucial role in the pathological changes of cerebral hemorrhage, which eventually leads to neuronal death (Kuramatsu et al., 2013; Taylor & Sansing, 2013; J. Wang, 2010).

Microglia are the earliest activated non‐neuronal cells after ICH, the primary source of molecules such as cytokines, chemokines, and proteases. Some molecules can induce secondary brain injury; others play a role in tissue repair. The activated microglia can differentiate into M1 and M2 phenotypes. M1 phenotype can produce tumor necrosis factors (TNF)‐α, interleukin (IL)‐1β, IL‐6, and other cytokines, which can aggravate the inflammatory reaction in the nervous system and result in nerve function damage (J. Wang, 2010). Moreover, the M2 phenotype can suppress the inflammatory response by secreting IL‐10.

Programmed cell death protein 1 (PD‐1) is a transmembrane glycoprotein on the surface of cytotoxic T lymphocytes. PD‐1 and its ligand, programmed cell death protein ligand 1 (PD‐L1), play an essential role in the pathogenesis of autoimmune diseases. The combination can play a negative feedback‐regulating effect on inflammation, suppress the immune response, and attenuate immune response (Martinic & Herrath, 2008). The PD‐L1 on B cells can inhibit the activation of T cells, microglia, or macrophages. Some studies have reported that the PD‐1/PD‐L1 pathway through intercellular interaction can negatively regulate inflammation response after stroke and play protective effects (Lin et al., 2012; Shi et al., 2015). The overexpression of PD‐1 and PD‐L1 after ICH can regulate inflammatory response and reduce secondary brain injury (Wu et al., 2017).

Electro‐nape‐acupuncture (ENA) is a kind of acupuncture therapy for treating neurological disorders, which is the product of the combination of traditional acupuncture technology and modern medical theory. Clinical studies have shown that ENA intervention can improve nerve function in ICH patients and promote the recovery of cognitive function (G. F. Wang et al., 2012, 2013). It also helps patients with tracheotomy improve ventilation function, remodel cough reflexes, and promote the recovery of lung infections (Jia et al., 2020). Our studies found that ENA intervention can significantly reduce cerebral edema caused by ICH, promote the metabolism of blood components in the hematoma lesions, and thus help ICH rats to restore neurological function and relieve secondary brain injury.

Given the limited treatment options available for secondary brain injury resulting from ICH, ENA can serve as a valuable adjunctive approach in ICH treatment. However, the limited application of ENA arises from both the unclear mechanism of ENA and the significant number of critically ill and comatose patients with ICH who are difficult to acupuncture on the neck. As a result, there are inherent limitations in its widespread use. Therefore, it is imperative to investigate the regulatory mechanisms underlying ENA intervention in mitigating secondary brain injury and establish a solid theoretical foundation for its clinical application. The study observed the effects of ENA on microglia differentiation in the acute stage of ICH, analyzing the changes in the PD‐1/PD‐L1 pathway. To investigate the possible mechanisms by which ENA intervention suppresses inflammatory responses in the acute stage of ICH.

## 2 MATERIALS AND METHODS

### 2.1 Experimental animals

Healthy male Sprague‐Dawley rats at 8 weeks of age (300 ± 10 g) were purchased from Beijing Vital River Laboratory Animal Technology Co. Ltd. and housed under specific pathogen free (SPF) conditions in a 12/12‐h light/dark cycle room with controlled temperature (22 ± 3°C) and relative humidity (40%−50%) in the Experimental animal center of Capital Medical University. All the animal experiments were performed under the approval of the Animal Experiments and Experimental Animal Welfare Committee of Capital Medical University (AEEI‐2018‐143).

### 2.2 ICH model establishment and groups

The rats were anesthetized with 3% isoflurane by a small animal anesthesia machine (R550; RWD Life Science). More than 100 μL blood was withdrawn from the tail veins and quickly transferred into a 100‐μL microsyringe (LPM‐S‐100; Gaoge). Then, the rat was placed on the stereotaxic instruments (68526; RWD Life Science), and nose clamp and ear bars were used to secure the rat head. By exposing the skull and using a microdrill (78001; RWD Life Science), a hole (Φ1 mm) was made on 0.5 mm posterior to the bregma and 3.5 mm lateral to the midline.

The syringe was slowly put through the burr hole to the target position (5.5 mm under the skull), and a syringe pump (legato 130; KD Scientific) was used to inject 30 μL of autologous blood at a rate of 10 μL/min into the caudate nucleus. Then, we waited for 10 min, and the remaining blood was injected at the same rate. After the infusion, the needle was kept in place for 10 min and withdrawn slowly. The hole was sealed with bone wax (Johnson & Johnson), and the wound was sutured after disinfection. The rats were placed in cages and given adequate feed and water. The rats' body temperature was maintained at 37°C.

Animals of successful ICH model were randomly grouped into the model group (*n* = 9) and ENA group (*n* = 9) via random number generation by computer. In the sham group (*n* = 9), the syringe was put in the same position without pump autologous blood. The ENA group was given ENA intervention, and the model and sham group rats were not given any intervention.

### 2.3 ENA intervention

The ICH rats were given ENA intervention once a day for 3 days, which began on the first day after surgery. Both sides neck acupoints of *Fengchi* (GB20) and the fourth cervical *Jiaji* (EX‐B2) acupoints were selected for acupuncture. The locations are according to the rat acupoint map.

Acupuncture needles were inserted into *Fengchi* and cervical *Jiaji*. The positive poles of the electronic acupuncture instrument (SDZ‐V; Hwato) were connected to two‐side *Fengchi*, and the negative poles were connected to two‐side cervical *Jiaji*. The rarefaction waveform with the voltage of 1 V and frequency of 1 Hz was set for 15 min.

### 2.4 Neurological deficiency

The beam walking test was used to evaluate the neurological function by measuring the motor coordination and balance of rats when passing the narrow beams at 24 and 72 h after surgery. The number of times the hindpaws slipped off the beam was recorded to evaluate the recovery of motor function (Tamakoshi et al., 2014).

### 2.5 Hematoxylin‐eosin staining

Hematoxylin‐eosin (HE) staining was used to observe the arrangement and karyopyknosis of nerve cells, inflammatory cell infiltration, and hematoma formation in rat brain tissues. The collected brains were fixed with 4% paraformaldehyde and then embedded with paraffin; then, the paraffin tissues were sectioned into 5‐μm slices. After paraffin sections dewaxing and washing, they were stained with hematoxylin staining for 5 min, differentiated with 1% hydrochloric acid alcohol for 1–3 s, blued in 1% aqua ammonia, and then stained with eosin for 30 s. Finally, sections were analyzed under an optical microscope (Sumbria et al., 2016).

### 2.6 Western blot

The tissues around the lesion were lysed by RIPA buffer with protease inhibitor cocktail (Roche, Switzerland). After determining the concentration of protein by BCA protein detection, it was separated by sodium dodecyl sulfate‐polyacrylamide gel electrophoresis and electrophoretically transferred to polyvinylidene difluoride (PVDF) membranes.

After blocking in 5% non‐fat milk, the PVDF membranes were incubated with the primary antibodies of PD‐1 (1:500, OM287612; Omnimabs), PD‐L1 (1:500, OM287625; Omnimabs), CD86 (1:1000, OM629693; Omnimabs), CD206 (1:1000, 18704‐1‐AP; Proteintech), and β‐actin (1:1000, TA‐09; ZSGB) overnight at 4°C. The membranes were incubated with secondary antibodies (goat anti‐rabbit/mouse IgG [H+L], 111‐035‐003/115‐035‐003; Jackson) after washing. Protein bands were detected by gel chemiluminescence imaging analysis system and analyzed by using Image J.

### 2.7 Immunofluorescence staining

Immunofluorescence staining was applied to observe protein expression of PD‐1 and PD‐L1. After paraffin sections dewaxing, antigen was recovered. The sections were incubated with primary antibodies of PD‐1 (1:200, OM287612; Omnimabs) and PD‐L1 (1:200, OM287625; Omnimabs) for 48 h. After rewarming and washing, the secondary antibodies (Alexa Fluor 488/TRITC, 1:400, Abcam, ab150109/ab6799). Lastly, DAPI (SouthernBiotech, 0100–20) was used to stain the cell nucleus and cover the coverslips.

Microglia differentiation was assessed by calculating the numbers of CD86^+^/CD11b^+^ and CD206^+^/CD11b^+^ cells around the lesion regions. Double staining sections were incubated with mixed primary antibodies which include CD86 (1:500, OM629693; Omnimabs) or CD206 (1:300, 18704‐1‐AP; Proteintech) plus CD11b (1:500, ab1211; Abcam). The number of positive cells was counted by another investigator who does not know about groups.

### 2.8 Real‐time PCR

Take 50 mg of frozen brain tissue, add 500 μL of Trizol, homogenize on ice until no visible tissue fragments, mix with chloroform and isopropanol, centrifuge and precipitate the RNA, and freeze it in liquid nitrogen. The primers gene for amplification are in Table 1.

**TABLE 1:** Primer sequences.

Receptor	Forward primer (5′–3′)	Reverse primer (5′–3′)
PD‐1	ATACGCCACCATTGTCTTCACTGA	AACTGTAGCTTGCACGTTCCTCTT
PD‐L1	GGAGAACCACACGGCTGAACTG	TCAAGCAGAAGAAGACGGTGAACC
TNF‐α	ATCCGAGATGTGGAACTGGC	CGATCACCCCGAAGTTCAGT
IL‐1β	TCAGGAAGGCAGTGTCACTCATTG	GGTCAGACAGCACGAGGCATT
IL‐6	TTGCCTTCTTGGGACTGATG	ATACTGGTCTGTTGTGGGTGGT
IL‐10	GCTGGACAACATACTGCTGACAGA	CTTCACCTGCTCCACTGCCTTG
β‐Actin	GAAGTGTGACGTTGACATCCG	GCCTAGAAGCATTTGCGGTG

Abbreviations: IL, interleukin; PD‐1, programmed cell death protein 1; PD‐L1, programmed cell death protein ligand 1; TNF, tumor necrosis factor.

For RNA expression detection, the One‐Step qPCR kit (Toyobo Life Science Inc.) and SYBR Green method in CFX Connect Real‐Time PCR System (Bio‐Rad) was used for analysis. The sample in the CFX Connect Real‐Time PCR machine was put, and β‐actin as an internal control was set. Amplification conditions were as follows: 95°C for 5 min denaturation, 95°C for 10 s and 60°C for 30 s fluorescence detection, for a total of 45 cycles. After amplification, the CT values of the gene and the internal control were exported for statistical analysis.

### 2.9 Enzyme‐linked immunosorbent assay

The tissue around the lesion was homogenized by using a homogenizer and centrifuged under 3000 rpm at 4°C for 20 min, and the supernatant fluid was collected. The enzyme‐linked immunosorbent assay (ELISA) kits (Neobioscience) were used to calculate the total protein concentration of supernatant fluid in sample tissue and measure the expression levels of inflammatory cytokines (TNF‐α, IL‐1β, IL‐6, IL‐10) which according to the specification.

### 2.10 Statistical methods

The experimental data were analyzed by Prism 8 (GraphPad) and expressed with the mean ± SEM. The neurological scores and data at each time point were analyzed by repeated‐measures analysis of variance (ANOVA). Other data of three groups were analyzed by one‐way ANOVA with Bonferroni post hoc test. When *p*‐values < .05, the differences were considered statistically significant.

## 3 RESULTS

### 3.1 ENA alleviated neurological deficiency in the acute phase of ICH rats

**FIGURE 1:**
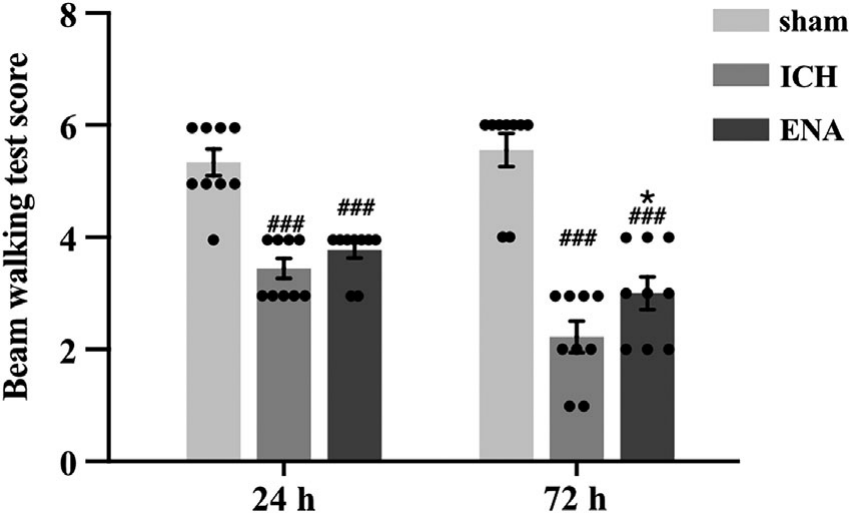
Electro‐nape‐acupuncture (ENA) intervention alleviated the beam walking test score at 24 h and 72 h after surgery (*n* = 9). Data are expressed as mean ± SEM, compared with the sham group, ###*p* < .001; compared with the intracerebral hemorrhage (ICH) group, *p < .05.

For evaluating the intervention effect of ENA on the acute phase of ICH rats, neurological function scores were assessed by beam walking at 24 and 72 h after surgery. Compared with the sham group, rats in the ICH group rats hindpaws slip from the surface of the beam many times at 24 h after the operation, and the nerve function damage was obvious (*p* < .001). Seventy‐two hours after the operation, the ICH rats were unable to pass the beam and stayed in place, and the nerve function damage was aggravated, but this trend was alleviated in the ENA group in which the 72‐h scores decreased less than the ICH group (*p* < .05). The results show that ENA can promote the recovery of neurological function in the acute phase of ICH rats (Figure 1).

### 3.2 ENA reduced acute phase inflammatory cells infiltration and protected nerve cells

**FIGURE 2:**
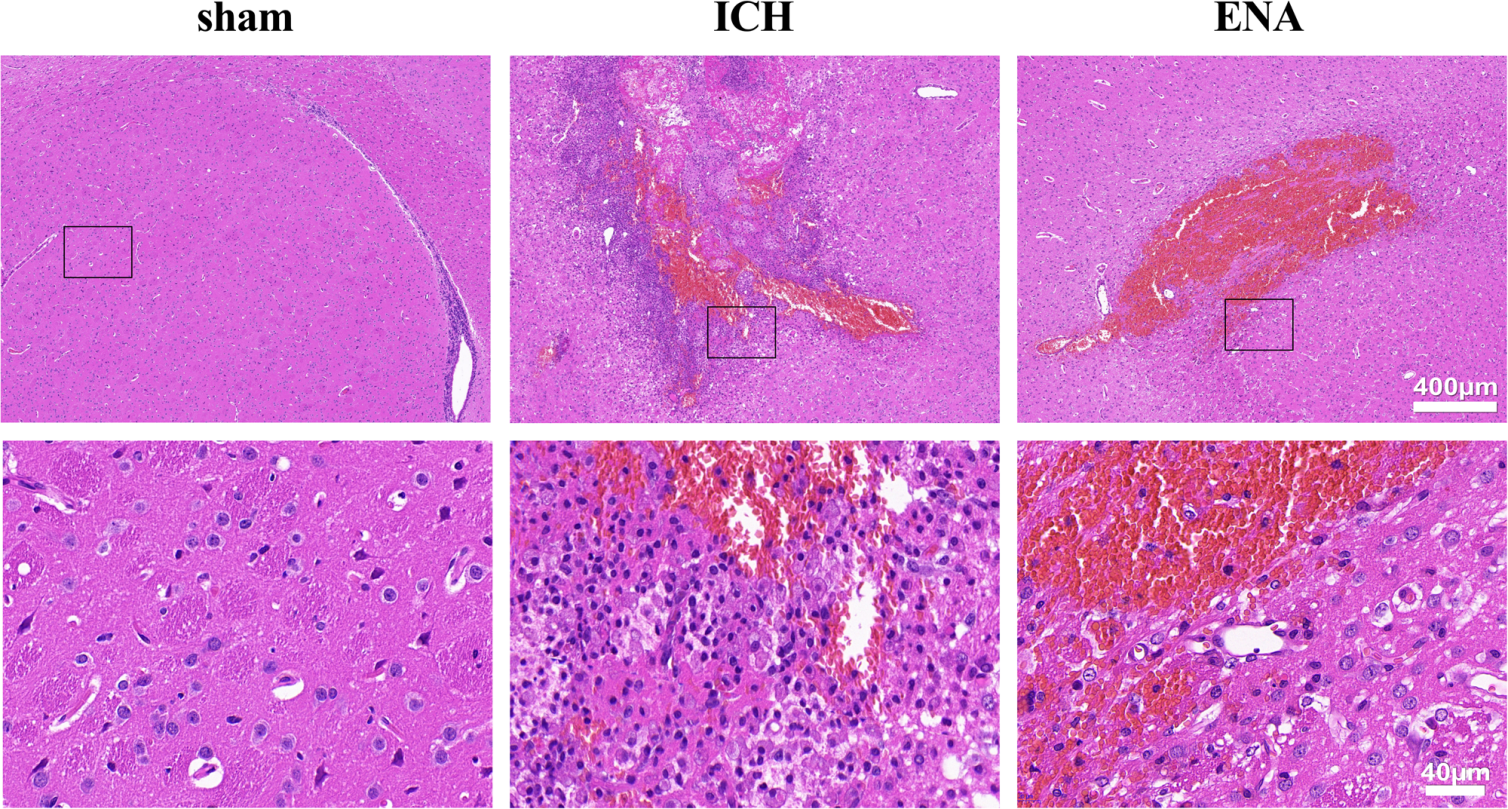
Electro‐nape‐acupuncture (ENA) intervention reduced inflammatory cells infiltration and protected nerve cells in intracerebral hemorrhage (ICH) rats. (Inflammatory cells infiltration and cell necrosis in the caudate nucleus were observed by hematoxylin‐eosin (HE) staining. Scale bars:40 μm).

To study the effects of ENA on nerve cells and inflammatory cell infiltration, we used HE staining for observation. Compared with the sham group, the hematoma lesions were observed in the caudate nucleus of the ICH group after 72 h, which damaged brain tissue structure, disordered the arrangement of peripheral nerve cells, and widened tissue gaps. The nucleus changes including karyopyknosis, thick dyeing, and a large number of inflammatory cell infiltration could be observed. After ENA intervention, the gap between nerve cells, the number of karyopyknosis, thick dyeing cells, and inflammatory infiltration was decreased significantly than the sham group. It is suggested that ENA can alleviate the injury of nerve cells, inhibit inflammatory response after, and reduce cerebral edema ICH (Figure 2).

### 3.3 ENA promoted the expression of PD‐1 around the lesion area in the acute phase of ICH rats

**FIGURE 3:**
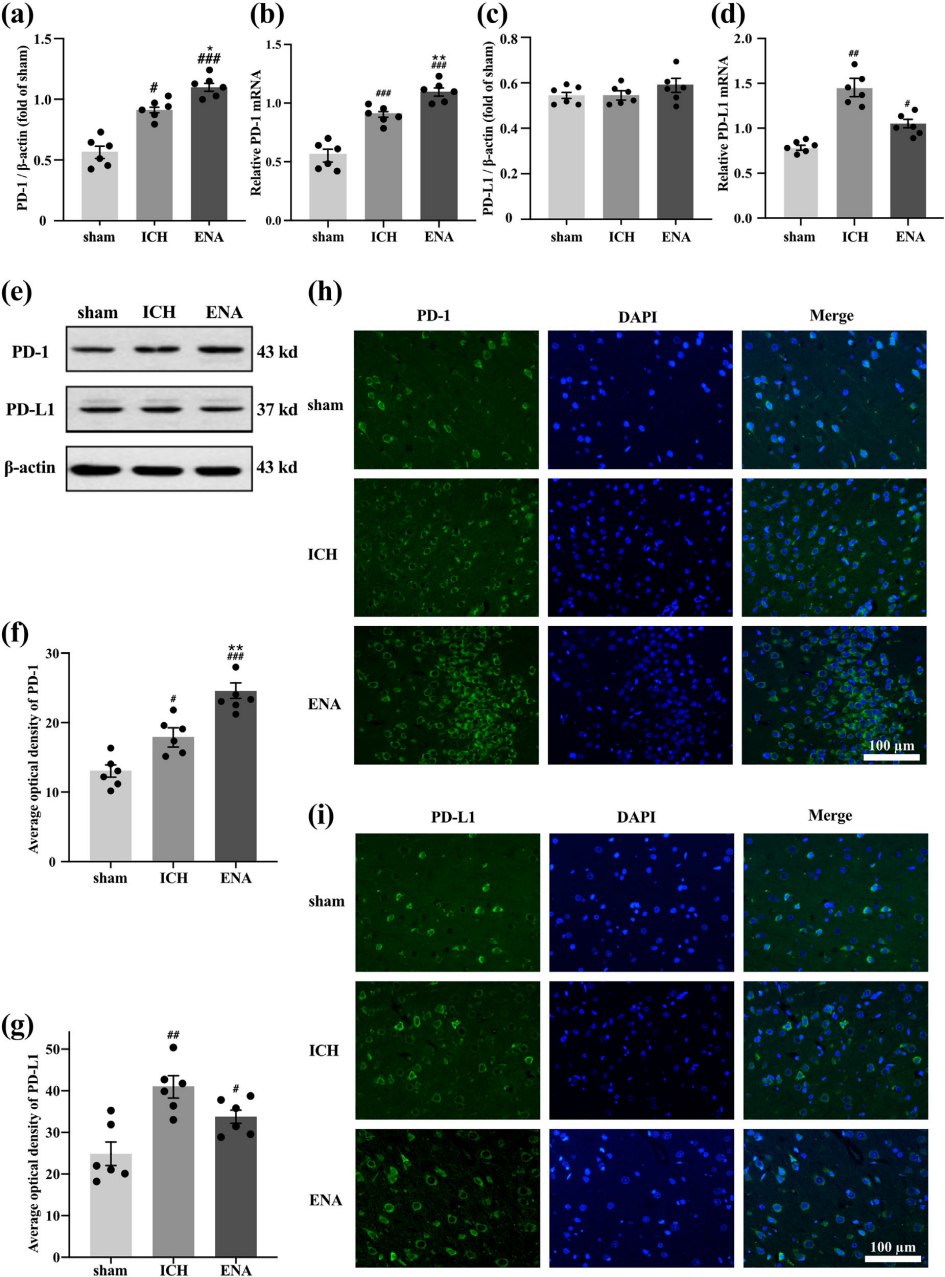
Electro‐nape‐acupuncture (ENA) promoted the expression of programmed cell death protein 1 (PD‐1) around the lesion area in intracerebral hemorrhage (ICH) rats (*n* = 6). (a and b) Analysis of PD‐1 protein and mRNA around lesion area. (c and d) Analysis of programmed cell death protein ligand 1 (PD‐L1) protein and mRNA around lesion area. (e) The representative western blot bands of PD‐1 and PD‐L1 around lesion area. (f and g) Quantification of the average optical density (AOD) of PD1+ and PD‐L1+ cells around lesion area. (h and i) PD1 and PD‐L1 cells around the lesion area were labeled by immunofluorescence (*n* = 3, each sample was chosen two fields of view). Scale bars: 100 μm. Data are presented as mean ± SEM, compared with the sham group, ###*p* < .001, ##*p* < .01, #*p* < .05; compared with the ICH group, **p < .01, *p < .05.

The expression of PD‐1 and PD‐L1 plays a key role in the inflammatory response after ICH; therefore, the study investigated whether ENA intervention affects the expression of PD‐1 and PD‐L1. The expression level of PD‐1 protein and mRNA in the ICH group was higher than it was in the sham group 72 h after surgery (*p* < .05, *p* < .001). After ENA intervention, the expression of PD‐1 increased significantly compared with the ICH group (*p* < .05, *p* < .01), which indicates that the expression of PD‐1 in brain tissue damaged by ICH is elevated, and electric acupuncture intervention can promote PD‐1 expression in the lesion. Similarly, both the model group and ENA group showed a significant increase in the expression of PD‐L1 mRNA compared to the sham group after modeling (*p* < .01, *p* < .001), with the model group displaying a more prominent increase than the ENA group. However, these mRNA results were inconsistent with protein expression results, indicating that ENA may have an impact on the expression of PD‐L1, but with a delayed effect compared to PD‐1 expression (Figure 3a–d).

The results of immunofluorescence staining showed that in the acute phase of ICH, the average optical density (AOD) of PD‐1^+^ and PD‐L1^+^ in ICH was higher than that in the sham group (*p* < .05, *p* < .01). In the ENA group, the AOD of PD‐1^+^ was significantly higher than that in the ICH group (*p* < .01), and there was a tendency to migrate around the lesion. In the expression of PD‐L1, the positive expression of PD‐L1 in the ICH group and ENA group increased, but the difference in AOD between the two groups was not significant (Figure 3f–i).

These results indicate that ENA intervention reduces the inflammatory response in the acute phase of ICH, which could be related to the regulation of PD‐1 expression.

### 3.4 ENA inhibited the differentiation of microglia around the lesion in the acute phase of ICH rats

**FIGURE 4:**
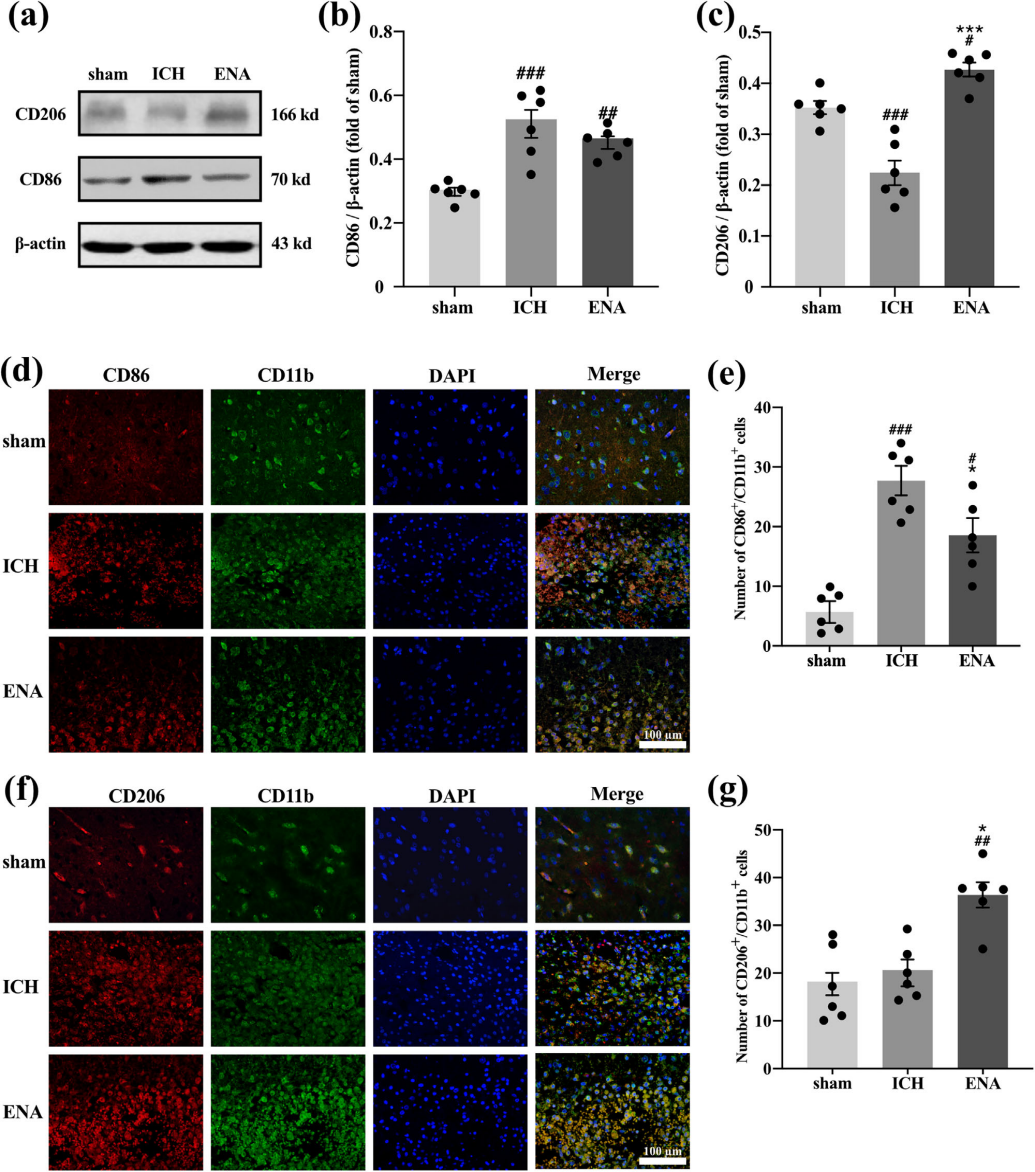
Electro‐nape‐acupuncture (ENA) regulated the expression of CD86 and CD206 protein around the lesion area in intracerebral hemorrhage (ICH) rats. (a–c) Representative western blot bands and analysis of CD86 and CD206 around the lesion area (*n* = 6). (d and f) Immunofluorescence of CD86+/CD11b+ and CD206+/CD11b+ cells in the lesion area (*n* = 3, each sample was calculated from two separate fields of view). Scale bars: 100 μm. (e and g) Quantification of the number of CD86+/CD11b+ and CD206+/CD11b+ cells around the lesion area in per field of vision. Western blot data are presented as mean ± SEM, compared with the sham group, ###*p* < .001, ##*p* < .01, #*p* < .05; compared with the ICH group, ***p < .001, *p < .05.

To observe the effect of ENA intervention on the differentiation of microglia, the experiment detected the marker proteins of M1 (CD86) or M2 (CD206) around the lesion in ICH rats. The M1 phenotype marker protein level in the ICH group was higher than that in the sham group (*p* < .001). However, the expression level of the sham group did not decrease significantly after ENA intervention. In contrast, the trend of CD206 expression in the ICH group was opposite to that in the ENA group (*p* < .001), which increased in the ENA group and decreased in the ICH group. The result suggested that the intervention of ENA could increase the expression of CD206 and play a protective role (Figure 4a–c).

Subsequently, we further investigated the differentiation of microglia around the lesion. Brain sections were double‐stained for CD86/CD11b or CD206/CD11b by immunofluorescence. Seventy‐two hours post‐operation, immunofluorescence double staining was used to observe the differentiation of microglia around the lesion. CD86 and CD206 were stained with red fluorescence. The marker proteins of microglia, CD11b, were stained with green fluorescence.

The staining results showed that the number of CD86^+^/CD11b^+^ cells around the lesion in the ICH group and ENA group was significantly higher than that in the sham group (in the caudate nucleus) (*p* < .001, *p* < .05). Compared with the ICH group, the number of CD86^+^/CD11b^+^ cells in the ENA group was lower than in the ICH group (*p* < .05). It is suggested that after hemorrhage, the hematoma could promote the differentiation of microglia to the M1 phenotype, then stimulate the inflammatory response, and ENA intervention can inhibit this process (Figure 4d,e). In the expression marker of M2 microglia, the number of CD206^+^/CD11b^+^ cells around the lesion in the ENA group was significantly higher than that in the ICH group (*p* < .05). It demonstrated that ENA intervention could promote microglia differentiate to M2 phenotype, inhibit the inflammatory response, and exert a neuroprotective effect (Figure 4f–g).

Notably, we could observe the migration of microglia around and inside the lesion after hemorrhage, which leads to the distribution of microglia around the lesion unevenly in immunofluorescence. At the same time, both of the non‐specific bindings of blood components in hematoma to antibodies and the lysis of microglia after phagocytosis of red blood cells interfere with the statistics of the AOD. Therefore, the experimental program still needs to be optimized.

**FIGURE 5:**
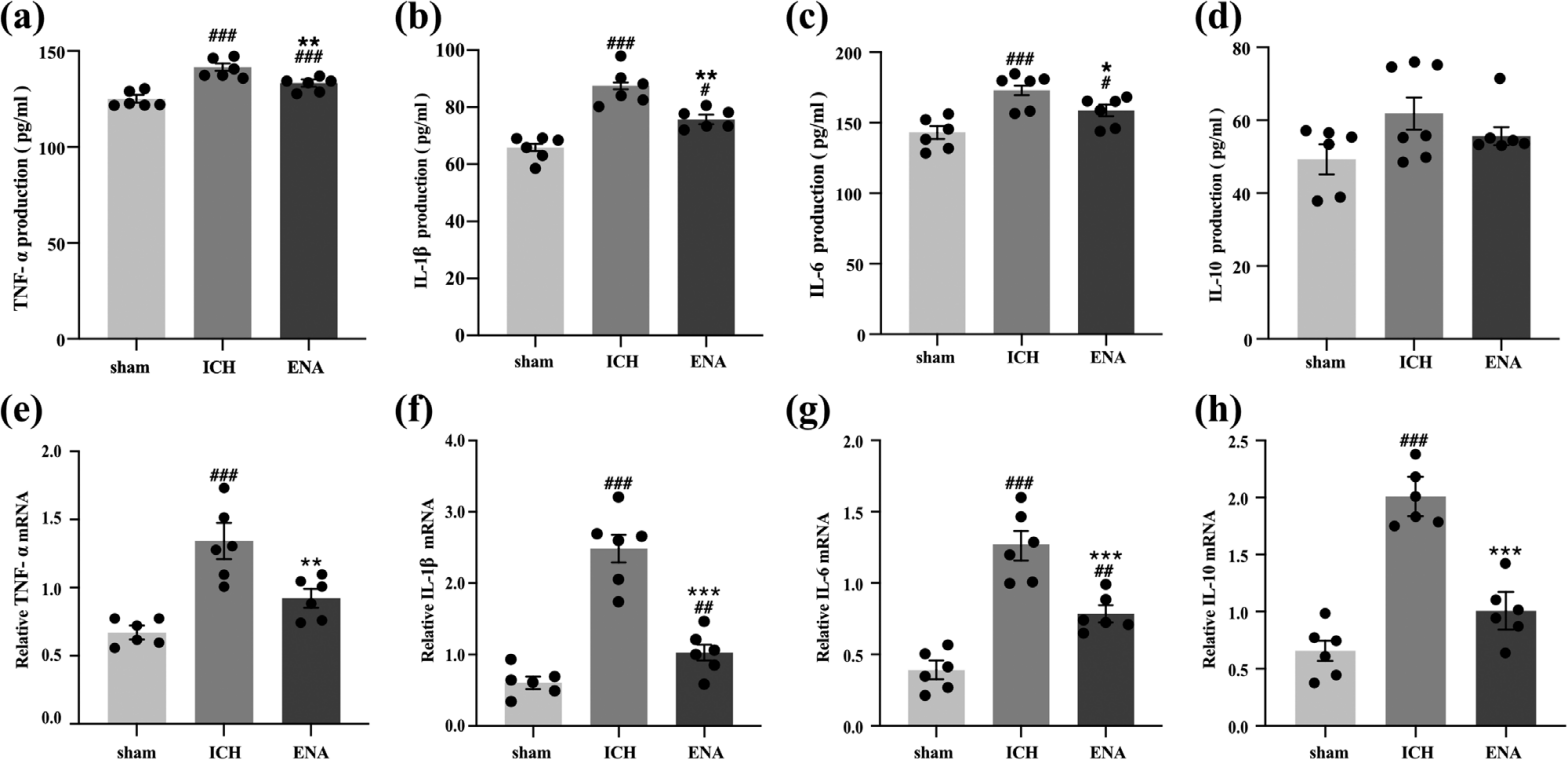
The expression of cytokine around the lesion area in intracerebral hemorrhage (ICH) rats were detected by enzyme‐linked immunosorbent assay (ELISA) and reverse transcription polymerase chain reaction (RT‐PCR) (*n* = 6). Data are presented as mean ± SEM, compared with the sham group, ###*p* < .001, ##*p* < .01,#*p* < .05; compared with the ICH group, **p < .001, *p < .05.

Microglia can secrete corresponding pro‐ or anti‐inflammatory cytokines after differentiation. In the experiment, ELISA and RT‐PCR were used to evaluate the regulation of ENA intervention on microglia. Results showed that the expression levels of pro‐inflammatory cytokines TNF‐α, IL‐1β, and IL‐6 in the ICH group were higher than those in the sham group. ENA intervention could inhibit the increase of inflammatory cytokines in brain tissue after ICH. In addition, the expression level of anti‐inflammatory, IL‐10, secreted by M2 phenotype microglia, was no significant difference between the three groups, which was not consistent with the PCR results (Figure 5).

## 4 DISCUSSION

In the acute phase of ICH, the overactivation of inflammatory response is inhibited by the high expression of PD‐1 and PD‐L1 (Wu et al., 2017). By setting agonists and inhibitor groups for comparison, researchers found that increasing PD‐1 and PD‐L1 expression can reduce the degree of cerebral injuries. Instead, cerebral injuries increase progressively. The study also found that PD‐1 can inhibit M1 microglia differentiation from inhibiting post‐ICH inflammatory reaction by regulating the phosphorylation of transducer and activator of transcription 1 (STAT‐1). PD‐L1 is expressed on the surface of microglia, macrophages, astrocytes, and other non‐hematopoietic cells (Phares et al., 2009; Schreiner et al., 2008). Studies have shown that the expression of PD‐L1 on microglia increases during the inflammation of the central nervous system (Duncan & Miller, 2011). It binds with PD‐1 on T‐cells and can relieve the proliferation of T‐cells, cytokine secretion, and cytotoxicity, and thus reducing the inflammatory response (Barber et al., 2006; Jeong et al., 2008; Latchman et al., 2004). The mechanism of other molecules that regulate the activation of microglia by the PD‐1/PD‐L1 pathway requires further study and confirmation.

In western blot and immunofluorescent staining, we have observed that ENA can increase proteins and positive cell expression of PD‐1 in tissues around lesions after ICH to inhibit overactive inflammatory reactions. However, the difference of protein and immunofluorescent in PD‐L1 was insignificant between the model and ENA group. Therefore, the experimental results suggest that ENA can reduce the inflammatory response after brain injury by increasing PD‐1 expression. The difference in PD‐L1 may be related to protein expression. Research suggests that PD‐1 protein level is at peak on 24 h after ICH and lasts 72 h. PD‐L1 has the same peak, but 72 h has shown a significant downward (G. F. Wang et al., 2013). Therefore, only one point in time after ICH is not suitable for observing the regulation effect of ENA for PD‐L1. Meanwhile, elevated expression of PD‐1 around the lesions may be achieved by increasing surface receptor expression or promoting the migration of PD‐1‐positive cells to the lesions. This presupposition needs a more thought‐out design of experiments to identify them.

Microglia is a phagocyte that is present in the brain. This process that microglia differentiate into pro‐inflammatory M1 phenotype or anti‐inflammatory M2 phenotype is called differentiation (Fumagalli et al., 2015; Xiong et al., 2016). Microglia activation can be identified by immunohistochemical/ immunofluorescent staining in ICH rat brain slices. Typical markers of microglia include keratin sulfate, CD11b, ionized calcium‐binding adapter molecule 1 (Iba‐1), and CD68. A study has shown that CD11b is expressed only on microglia in the uninjured brain. When ICH occurs, due to the blood‐brain barrier (BBB) being damaged and cell immersion, CD11b is expressed on mononucleocytes and neutrophils, so labeling small glial cells with CD11b alone does not make a good distinction between expression states in brain tissue, it needs to be verified by multiple labeling by selecting different markers (Hammond et al., 2012; Zhao et al., 2015).

At 24 and 72 h after hemorrhage, M1 phenotype markers such as CD16, CD32, and inducible nitric oxide synthase (iNOS) are highly expressed, suggesting that M1 microglia differentiation occurs in the early phase of ICH and promotes the inflammatory response (Lan et al., 2017). Studies have found that nuclear factor‐κB (NF‐κB) in the tissues surrounding hematoma lesion will be activated 13–48 h after hemorrhage, and the expression levels of IL‐1β and TNF‐α increase within 1 day (Wu et al., 2010; Zhang et al., 2014). In ICH animal model induced by collagenase and autologous blood injection, mRNA levels of the pro‐inflammatory markers IL‐1β, IL‐6, TNF, and iNOS were elevated in the brains during the ICH acute phase (Liesz et al., 2011; Matsushita et al., 2014; Yang et al., 2013). The expression level usually starts to increase at 3 h in the early stage of ICH, peaks on the third day after bleeding, and returns to the average level on the seventh day (Lin et al., 2012; Wasserman et al., 2007). M2 phenotype microglia play an important role in phagocytosis and removal of toxic substances in neurological diseases (Chen & Trapp, 2016) Both clinical and animal experiments have shown that IL‐10 expression levels increase in blood and brain tissue after ICH (Gao et al., 2014; Shi et al., 2015). In vitro, IL‐10 promotes the differentiation of microglia and macrophages into the M2c phenotype and essential the phagocytosis of monocytes. A study has found that the expression of IL‐10 did not change significantly in the autogenous blood intracerebral hemorrhage model (Taylor et al., 2017).

The increased concentration of TNF‐α in the acute phase of ICH induces the expression of intercellular cell adhesion molecule (ICAM), which produces more inflammatory cytokines, destroys the BBB, and promotes cytokines release, like platelet‐activating factor (PAF) and clotting factor VIII, which can increase blood viscosity and accelerate the cell apoptosis in the brain (Tschoe et al., 2020). IL‐1β expression increases vascular endothelial injury (Holmin & Mathiesen, 2000). IL‐6 promotes the migration of inflammatory cells from blood vessels to brain tissues, releases reactive oxygen species (ROS) and elastase, aggravates brain edema, and promotes nerve cell necrosis or apoptosis (Holmin & Mathiesen, 2000; Yao et al., 2014; Zhang et al., 1995). As an anti‐inflammatory factor, IL‐10 inhibits the secretion of inflammatory cytokines and plays a protective role after ICH (Berghmans & Al‐Obeidan et al., 2019; Bharhani et al., 2006; Stein et al., 2011). Our study found that the intervention of ENA reduces the expression of TNF‐α, IL‐1β, and IL‐6 in the tissues around the hematoma lesion after hemorrhage, thereby reducing the inflammatory injury. However, the change of IL‐10 in the test result is not apparent, which is consistent with the results of previous studies, so in the next experiment, the inhibitory effect of M2 microglia by other downstream molecules needs to be analyzed (Taylor et al., 2017).

The *fēng chí* of gallbladder channel is an important point for unblocking the collaterals and relieving pain. The extra point (*gòng xiě*) is also located on the gallbladder channel which is the main treatment for stroke. Three yang channels of the hand and foot, as well as *du mai* and *ren mai*, all reach the head. The foot *taiyang* channel enters the brain directly, while the *du mai* and *ren mai* are connected to the brain. The other five yang channels pass through the neck and intersect with the *du mai* at the *dà zhuī* point to indirectly contact the brain. The foot *shaoyang* gallbladder channel runs along the lateral of the head, and the foot *jueyin* liver channel runs from the forehead to the top of the head, and they are the interior‐exterior relationship between them. The anatomical structure of *fēng chí* and extra point (*gòng xiě*) in the nape shows that the occipital artery and vein are located superficially and that the vertebral artery is located deeper. Acupuncture both acupoints, combined with stimulation using dilatational waves, can effectively enhance relaxation and contraction functions of vascular smooth muscle, thereby improving spasticity. Additionally, these acupoints have been found to alleviate tension in muscle groups such as the superficial trapezius muscle, regulate circulation obstruction caused by tissue compression, reduce circulation resistance, accelerate blood flow dynamics, improve head blood supply, and facilitate metabolic processes for pathological products. Electroacupuncture can also help to produce nerve impulses and stimulates the central nerve to re‐establish damaged nerve reflexes, thereby improving nerve function after ICH. Thus, these two acupoints can be used to treat ICH.

According to the study, intervention with ENA has been shown to increase PD‐1 expression and reduce inflammatory response during the acute phase of cerebral hemorrhage. Moreover, it also increases protective M2 microglia expression while decreasing pro‐inflammatory factor release, thereby reducing secondary damage. Considering these findings along with previous research studies, ENA could be further studied as an effective intervention for inhibiting secondary brain injury after ICH and protecting nerve cells (Liu et al., 2020; Shen et al., 2019; X. Wang et al., 2016; Zhang et al., 2021).

## AUTHOR CONTRIBUTIONS

H.C. and Y.L. designed the study, performed of ICH model and molecular experiments. X.Z.,W.G. and R.D. performed animal experiments and analyzed data. L.Z. and H.Y. provided resources and edited the manuscript. S.Z. and H.C. conceived the project, provided funding, and edited the manuscript.

## CONFLICT OF INTEREST STATEMENT

The authors declare no conflict of interest.

### PEER REVIEW

The peer review history for this article is available at https://publons.com/publon/10.1002/brb3.3229.

## Data Availability

The datasets used and/or analyzed during the current study are available from the corresponding author on reasonable request.
